# Evolving epidemiology and antimicrobial resistance in spontaneous bacterial peritonitis: a two-year observational study

**DOI:** 10.1186/1471-2334-14-287

**Published:** 2014-05-23

**Authors:** Lionel Piroth, André Pechinot, Vincent Di Martino, Yves Hansmann, Alain Putot, Isabelle Patry, Tahar Hadou, Benoit Jaulhac, Catherine Chirouze, Christian Rabaud, Alain Lozniewski, Catherine Neuwirth, Pascal Chavanet, Anne Minello

**Affiliations:** 1 Département d’Infectiologie, CHU Dijon, Dijon, France; 2 Université de Bourgogne, Dijon, France; 3 Laboratoire de Bactériologie, CHU Dijon, France; 4 Service d’Hépato-Gastro-Entérologie, CHU Besançon, France; 5 Service de Maladies Infectieuses et Tropicales, CHU Strasbourg, Strasbourg, France; 6 Laboratoire de Bactériologie, CHU Besançon, France; 7 Laboratoire de Bactériologie, CHU Nancy, France; 8 Laboratoire de Bactériologie, CHU Strasbourg, France; 9 Service de Maladies Infectieuses et Tropicales, CHU Besançon, France; 10 Service de Maladies Infectieuses et Tropicales, CHU Nancy, France; 11 Service d’Hépato-Gastro-Entérologie, CHU Dijon, France

**Keywords:** Bacterascites, Spontaneous bacterial peritonitis, Epidemiology, Antibiotic susceptibility, Resistance

## Abstract

**Background:**

Current recommendations for empirical antimicrobial therapy in spontaneous bacterial peritonitis (SBP) are based on quite old trials. Since microbial epidemiology and the management of patients have changed, whether these recommendations are still appropriate must be confirmed.

**Methods:**

An observational study that exhaustively collected the clinical and biological data associated with positive ascitic fluid cultures was conducted in four French university hospitals in 2010–2011.

**Results:**

Two hundred and sixty-eight documented positive cultures were observed in 190 cirrhotic patients (median age 61.5 years, 58.5% Child score C). Of these, 57 were classified as confirmed SBP and 140 as confirmed bacterascites. The predominant flora was Gram-positive cocci, whatever the situation (SBP, bacterascites, nosocomial/health-care related or not). Enteroccocci (27.7% *E. faecium*) were isolated in 24% of the episodes, and in 48% from patients receiving quinolone prophylaxis. *E. coli* were susceptible to amoxicillin-clavulanate and to third-generation cephalosporins in 62.5% and 89.5% of cases, respectively. No single antibiotic allowed antimicrobial coverage of more than 60%. Only combinations such as amoxicillin + third-generation cephalosporin or cotrimoxazole allowed coverage close to 75-80% in non-nosocomial episodes. Combinations based on broader spectrum antibiotics should be considered for empirical therapy of nosocomial infections.

**Conclusions:**

Our study confirmed the changing spectrum of pathogens in SBP and bacterascites, and the need for more complex antibiotic strategies than those previously recommended. Our findings also underline the need for new clinical trials conducted in the current epidemiological context.

## Background

Spontaneous bacterial peritonitis (SBP), defined as an infection of ascites in the absence of a contiguous source of infection, is a frequent and severe complication in cirrhotic patients, with a significant risk of mortality, ranging initially from more than 80% in historical reports to 10-30% in the most recent studies [1-3]. Early diagnosis and early optimal treatment of these infections with appropriate antibiotics and the prevention of hepatorenal syndrome with albumin are required [4]. Current European and most other international guidelines recommend the use of a third-generation cephalosporin as the first choice, or amoxicillin-clavulanate acid or fluoroquinolones as an alternative choice [5-7]. These recommendations are based mainly on clinical trials that were very often conducted a decade or more ago, and on the assumption that *E. coli* would be involved in nearly half of the cases.

Several studies have pointed out changes in the epidemiology of the causative bacteria in SBP and bacterascites and in their susceptibility to antibiotics. In particular, the development of beta-lactamase enzymes, which confer resistance to clavulanate, or extended spectrum beta-lactamases in *Escherichia coli*[8]. The potential emergence of enterococci, methicillin-resistant *S. aureus*, or fluoroquinolone-resistant bacteria, following norfloxacin prophylaxis, is also a cause of concern since they may be associated with a higher risk of therapeutic failure [9].

There is thus a need for regular epidemiological studies. We previously conducted a retrospective multi-center study in North Eastern France, which showed the growing implication of Gram positive bacteria, in particular enterococci, and the increasing frequency of acquired antimicrobial resistance among some of the bacteria involved (in particular *E Coli*) [10].

A new 2-year observational study was conducted in the same area to confirm the changes in the spectrum of causative pathogens in SBP and bacterascites, their biological and clinical presentations, and therapeutic interventions and outcomes.

## Methods

### Study design

The study was conducted in four university hospitals in north-eastern France (Besançon, Dijon, Nancy and Strasbourg) between 1^st^ January 2010 and 31^st^ December 2011, in compliance with the Helsinki Declaration and the approval of IDD Ethics Committee. All the infectious agents isolated from peritoneal liquid in cirrhotic patients hospitalized during this period were systematically and prospectively recorded by the referent microbiologist of the participating hospital. There was no limitation regarding the indication of paracentesis, but infectious agents isolated from peritoneal dialysis or from secondary bacterial peritonitis were not included. When the same strain was isolated twice or more from the same patient within a month, only the first was considered.

### Microbiological and biological data

For each positive ascitic fluid culture, the biological characteristics of the ascitic fluid, as well as the microbiological characteristics of the infectious agent (s) isolated were recorded. Antibiotic susceptibility was assessed with antibiotic disks (Bio-Rad, Marnes-la –Coquette, France; or Vitek 2 System, Bio Mérieux, Tassin la demi-lune, France) according to the 2010–2011 updated recommendations of the CA-SFM (comité de l’Antibogramme de la Société Française de Microbiologie) (http://www.sfm-microbiologie.org). In the determination of the antimicrobial coverage of antibiotics, intermediate strains were considered resistant. Multi-drug resistant bacteria were defined as vancomycin-resistant enterococci, methicillin-resistant staphylococci, extended-spectrum betalactamase-producing Gram-negative bacteria, carbapenemase-producing Gram-negative bacteria, and Gram-negative bacteria resistant to multiple classes of antibiotics (i.e. at least three usually-active drugs from different classes, in particular penicillins + beta-lactamase inhibitors, antipseudomonal penicillins + beta-lactamase inhibitors, 2^nd^ generation cephalosporins, 3^rd^ generation cephalosporins, cephamycins, monobactams, aminoglycosides, folate pathway inhibitors, fosfomycin, fluoroquinolones).

SBP was defined as bacterial growth with an ascitic neutrophil count above 250/mm^3^, whereas bacterascites was defined as bacterial growth with an ascitic neutrophil count below this threshold [2,11]. When the ascitic neutrophil count was not available (because of immediate bedside inoculation into culture bottles and/or failure to count neutrophils), the cases were not classified into these two categories.

The infection was deemed nosocomial when the ascitic fluid paracentesis was performed after 48 hours of hospitalization. The infection was deemed related to health care when it was nosocomial and/or when it occurred in patients with previous repeated and/or recent hospitalization (<3 months) for medical care. Thus, though a nosocomial infection is always health-care related, a health-care related infection may be not nosocomial.

### Clinical data collection

The clinical presentation at the time of ascitic fluid paracentesis and the different antibiotics administered were collected by the local investigator for each positive ascitic fluid culture, in particular to ensure that no secondary peritonitis was suspected. The final outcome (i.e. in hospital survival) was also collected.

### Statistical analyses

Categorical variables were compared using Fisher’s exact and chi-square tests, and continuous variables using the Kruskall Wallis test. Analyses were performed in the whole group, in the subgroup with available ascitic neutrophil counts (allowing differentiation between SBP and bacterascites), and distinguishing between nosocomial or not, or health-care related or not. Since it cannot be excluded that coagulase-negative staphylococci were skin contaminants, even when associated with an ascitic neutrophil count above 250/mm^3^, sensitivity analyses were repeated after excluding the episodes involving coagulase-negative staphylococci. A backward step-by-step logistic regression was conducted for multivariate analysis of the factors associated with clinical outcome, including all of the factors associated in univariate analysis with a p value of less than 0.10.

All tests were two-sided and a p-value of less than 0.05 was considered statistically significant. All statistical analyses were done using Stata version 12.0.

## Results

### Study population

During the two-year study period, 6,220 samples of ascitic fluid from 1659 patients were analyzed. At least one infectious agent was found in 268 samples (4.3%). Of the 190 patients with at least one positive ascitic fluid culture, 80% were hospitalized in hepatology wards.

The median age of these patients at their first infection was 61.5 years (interquartile range (IQR): 53.4-69.8). There were 137 men and 53 women, most of whom presented severe cirrhosis (58.9% Child C, 36.3% Child B and 4.7% Child A) with a median Child score of 10 (IQR 8–12). The mean number of ascitic fluid samples in which an infectious agent was isolated was 1.41 +/-0.92 per patient, and the mean number of infectious agents isolated per positive sample was 1.29 +/- 0.77. Ten percent of these patients were on quinolone prophylaxis.

### Characteristics of the positive ascitic fluid cultures

#### *Biological and microbiological data*

The characteristics and the distribution of infectious agents involved in the 268 infections are shown in Table 1. Overall, Gram-positive cocci were found to be predominant (64.9%) followed by Gram-negative rods (33.9%), and 84.3% of the infections were monomicrobial. In all the plurimicrobial cases (15.7%, mainly involving only 2 infectious agents), secondary peritonitis was clinically excluded. MDR bacteria were involved in 34 episodes (13 vancomycin-resistant enterococci, 3 methicillin-resistant *Staphylococcus aureus*, 10 extended betalactamases-producing E Coli, 17 other multiresistant Gram-negative bacteria).

**Table 1:** **Distribution of the main infectious agents isolated in 268 ascitic fluid samples during the period 2010**–**2011**

	**Total (n = 268) (n,%)**	**Ascitic neutrophil count (median, IQR)**	**Non-nosocomial episodes (n = 109) (n,%)**	**Nosocomial episodes (n = 159) (n,%)**	**p**^**1**^	**SBP (n = 57) (n,%)**	**Bacterascites (n = 140) (n,%)**	**p**^**2**^	**With FQ prophylaxis (n = 27) (n,%)**	**Without FQ prophylaxis (n = 241) (n,%)**	**p**^**3**^
*Streptococcus* (*other than S. pneumoniae*)	33 (12.3%)	70 (21–150)	17 (15.6%)	16 (10.1%)	0.18	5 (8.8%)	18 (12.9%)	0.42	2 (7.4%)	31 (31.9%)	0.41
*Streptococcus pneumoniae*	4 (1.5%)	955 (200–1710)	4 (3.7%)	0 (0.0%)	0.01	1 (1.8%)	1 (0.7%)	0.51	0 (0.0%)	4 (1.7%)	0.50
*Enterococcus sp.*	65 (24 .3%)	75 (25–565)	15 (13.8%)	50 (31.4%)	0.0009	13 (22.8%)	31 (22.1%)	0.92	13 (48.1%)	52 (21.6%)	0.02
*Staphylococcus aureus*	7 (2.6%)	392 (18–815)	6 (5.5%)	1 (0.6%)	0.01	0 (0.0%)	0 (0.0%)	-	1 (3.7%)	6 (2.5%)	0.71
*Coagulase negative Staphylococcus*	73 (27.2%)	63 (8–155,5)	25 (22.9%)	48 (30.2%)	0.19	13 (22.8%)	47 (33.6%)	0.14	4 (14.8%)	69 (28.6%)	0.13
***Gram positive cocci***	174 (64.9%)	77 (10–310)	67 (61.5%)	107 (67.3%)	0.33	32 (56.1%)	91 (65.0%)	0.24	20 (74.7%)	154 (63.9%)	0.29
*Escherichia coli*	48 (17.9%)	129 (86–830)	20 (18.3%)	28 (17.6%)	0.88	15 (26.3%)	21 (15.0%)	0.06	3 (11.1%)	45 (18.7%)	0.33
*Klebsiella sp.Enterobacter sp. Serratia sp.*	29 (10.8%)	92 (58–549)	10 (9.2%)	19 (11.9%)	0.47	5 (8.8%)	10 (7.1%)	0.70	6 (22.2%)	23 (9.5%)	0.04
*Pseudomonas sp.*	9 (3.4%)	59 (44–670)	0 (0.0%)	9 (5.7%)	0.01	0 (0.0%)	5 (3.6%)	0.15	0 (0.0%)	9 (3.7%)	0.31
***Gram negative rods***	91 (34.0%)	100 (66–660)	32 (29.4%)	59 (37.1%)	0.18	23 (40.4%)	39 (27.9%)	0.09	9 (33.3%)	82 (34.0%)	0.94
Anaerobes	11 (4.1%)	112 (12–500)	8 (7.3%)	3 (1.9%)	0.03	2 (3.5%)	4 (2.9%)	0.81	0 (0.0%)	11 (4.6%)	0.26
Fungi	7 (2.6%)	52 (30–55)	2 (1.8%)	5 (3.1%)	0.51	0 (0.0%)	3 (2.1%)	0.27	0 (0.0%)	7 (2.9%)	0.37

Not all the infectious agents are listed in this table. Since several agents were isolated in the same sample of ascitic fluid, the total may exceed 100%.

^1^comparison between non-nosocomial and nosocomial episodes.

^2^comparison between SBP and bacterascites.

^3^comparison between episodes with and without fluoroquinolone (norfloxacine) prophylaxis.

Fifty-seven (21.3%) positive ascitic fluid samples were classified as confirmed SBP, 140 (52.2%) as confirmed bacterascites, and 71 (26.5%) could not be classified because of missing neutrophil counts (mainly because of immediate bedside inoculation into culture bottles without associated assessment of the neutrophil count). The SBP/bacterascites ratio was thus close to 1/2.5.

The median neutrophil count in the ascitic fluid was 92/mm^3^ (range 0–9700), and the median protein concentration was 14 g/L (range 2–125). In patients with confirmed SBP, the median neutrophil count in the ascitic fluid was 880/mm^3^ (range 270–9700), and the median protein concentration was 15 g/L (range 4–35). The median neutrophil count was higher in ascitic fluids with Gram-negative cocci than in those with Gram-positive ones (100 vs. 77/mm^3^, p = 0.003).

No significant difference in microbial distribution was observed between SBP and bacterascites, except for a non-significant trend towards a greater proportion of Gram negative rod infections in SBP (p = 0.09) even though such infections were still in the minority (40.4% vs. 56.1% Gram-positive cocci in SBP, respectively). MDR bacteria were more often involved in SBP than in bacterascites (19.3% vs. 7.1%, p = 0.006).

#### *Nosocomial and/or health care related episodes*

Ascitic liquid fluid paracentesis was performed within the first 48 hours of hospitalization for 109 (40.7%) episodes, thus defined as non-nosocomial. As 20 of these episodes followed repeated or recent hospitalizations, the number of potentially health-care related episodes was 179 (66.8%).

MDR bacteria were also significantly more often found in nosocomial (17.6% vs. 5.5% in non-nosocomial episodes, p = 0.002) and in healthcare-related episodes (16.2% vs. 5.6% in non-healthcare-related episodes, p = 0.006).

In all cases, Gram-positive cocci were predominant. However, pneumococci and *Staphylococcus aureus*, albeit rare, were more frequently isolated in non-nosocomial episodes, as were Gram-negative anaerobes, whereas enterococci were more frequently isolated in nosocomial and healthcare-related episodes. Indeed, the same distribution was observed in the 179 healthcare-related positive ascitic fluid samples as in the 89 non-healthcare-related samples, with a higher frequency of enterococci (31.3% vs. 10.1%, p = 0.0001 and of *Pseudomonas aeruginosa* (5.0% vs. 0.0%, p = 0.04), and a lower implication of pneumococci (0.0% vs. 4.5%, p = 0.01).

Enterococci (*E. faecium* in 18/65, 27.7%) were also isolated significantly more often in ascitic fluids from patients with previous quinolone prophylaxis (48.1% vs. 21.6% in those without quinolone prophylaxis, p = 0.04). The difference was even greater when considering SBP alone (50.0% in those with vs. 15.6% in those without quinolone prophylaxis, p = 0.02). KES (*Klebsiella*, *Enterobacter* and *Serratia* spp.) were also found more frequently in patients with previous quinolone prophylaxis (Table 1).

### Coverage of antimicrobials for positive ascitic fluid cultures

The effective coverage of antimicrobials for all of the infectious agents found in each positive ascitic fluid culture is shown in Table 2.

**Table 2:** **Susceptibility of the infectious agents found in 268 samples of ascitic fluid to different antimicrobials during the period 2010**-**2011**

	**Total (n = 268)**	**After exclusion of coagulase negative staphylococci (n = 195)**	**Non-nosocomial episodes (n = 109)**	**Nosocomial episodes (n = 159)**	**P**^**1**^	**SBP (n = 57)**	**Bacterascites (n = 140)**	***P***^***2***^
Amoxicillin	33.2%	45.6%	40.4%	28.3%	0.04	40.4%	33.3%	0.37
Amoxicillin + clavulanate	42.5%	53.3%	50.5%	38.1%	0.02	43.9%	44.3%	0.96
Broad-spectrum penicillin	35.4%	48.7%	38.5%	33.3%	0.38	40.4%	39.3%	0.89
Broad-spectrum penicillin + beta lactamase inhibitor	48.1%	64.1%	51.4%	45.9%	0.38	47.4%	50.7%	0.67
Third-generation cephalosporin	39.2%	39.0%	55.0%	28.3%	<0.0001	33.3%	42.1%	0.25
Fluoroquinolone	46.6%	46.7%	57.8%	39.0%	0.002	38.6%	47.1%	0.27
Aminoglycoside	50.0%	47.2%	56.9%	45.3%	0.06	47.4%	52.9%	0.48
Cotrimoxazole	42.2%	38.4%	53.2%	34.6%	0.002	29.8%	45.7%	0.04
Glycopeptide	57.8%	45.6%	60.6%	56.0%	0.45	50.9%	66.5%	0.04

Percentages indicate the in vitro antimicrobial efficacy on the infectious agent (s) isolated in positive ascitic fluid cultures.

(intermediate strains were considered non-susceptible).

^1^comparison between non-nosocomial and nosocomial episodes.

^2^comparison between SBP and bacterascites.

Many antibiotics used alone offer antimicrobial coverage close to 50%, but none allowed coverage higher than 60%. Excluding coagulase-negative staphylococci increased the expected coverage of most beta-lactams, except for third generation cephalosporins. The expected coverage of other antibiotics was unmodified, except that of glycopeptides, which was nearly 12% lower.

The coverage was similar for confirmed SBP and bacterascites, except for glycopeptides and cotrimoxazole, which showed lower coverage in SBP. Coverage for amoxicillin and amoxicillin-calvulanate was nearly 10% higher in non-nosocomial or non-healthcare–related than in nosocomial or healthcare-related) infections, respectively, and coverage for third-generation cephalosporins was more than 25% higher in non-nosocomial and non-healthcare-related infections.

The coverage of antimicrobials used alone or in combination in non-nosocomial infections (after exclusion of coagulase-negative staphylococci) or in confirmed SBP is shown in Table 3.

**Table 3:** ***In vitro *** **susceptibility to antibiotics alone** (**first row or column**) **or in combination** (**other rows**/**columns**) **on the infectious agents isolated either from non**-**nosocomial ascitic fluids after exclusion of coagulase negative staphylococci** (**upper right half**) **or from confirmed spontaneous bacterial peritonitis** (**including coagulase negative staphylococci** , **lower left half**)

**n = 84**		**amoxicillin**	**amoxicillin-clavulanate**	**Piperacillin-tazobactam**	**Third generation cephalosporin**	**Fluoro**-**quinolone**	**Aminoglycosides**	**Cotrimoxazole**	**Glycopeptide**
**n = 57**									
-		47.6%	63.1%	65.5%	54.8%	57.1%	50.0%	47.6%	50.0%
**Amoxicillin**	40.4%		-	-	77.4%	78.6%	82.1%	77.4%	69.0%
**Amoxicillin**-**clavulanate**	43.9%	-		-	-	81.0%	83.3%	81.0%	78.6%
**Piperacillin**-**tazobactam**	47.4%	-	-		-	79.8%	79.8%	78.6%	85.7%
**Third generation cephalosporin**	33.3%	56.1%	-	-		72.6%	69.0%	69.0%	84.5%
**Fluoroquinolone**	38.6%	61.4%	61.4%	61.4%	49.1%		-	65.5%	84.5%
**Aminoglycosides**	47.4%	73.7%	73.7%	68.4%	56.1%	-		66.7%	-
**Cotrimoxazole**	29.8%	57.9%	57.9%	57.9%	45.6%	49.1%	47.3%		78.6%
**Glycopeptide**	50.9%	71.9%	71.9%	80.7%	77.2%	84.2%	-	73.3%	

Combinations with in vitro coverage above 75% are in grey.

Most frequently found bacteria isolated from ascitic fluid samples (n = 268): coagulase negative staphylococci (n = 73): enterococci (n = 65), *Escherichia coli* (n = 48), streptococci (n = 32), and KES (*Klebsiella*, *Enterobacter*, *Serratia*) bacteria (n = 29).

Most frequently found bacteria isolated from non-nosocomial ascitic fluid samples (n = 109): coagulase negative staphylococci (n = 24), *Escherichia coli* (n = 20), streptococci (n = 17), enterococci (n = 16), and KES (*Klebsiella*, *Enterobacter*, *Serratia*) bacteria (n = 10).

Most frequently found bacteria isolated from confirmed SBP (n = 57): *Escherichia coli* (n = 15), coagulase negative staphylococci (n = 13), enterococci (n = 13), streptococci (n = 7), and KES (*Klebsiella*, *Enterobacter*, *Serratia*) bacteria (n = 5).

### Clinical outcome

Fifty-one patients (26.8%) died, 119 (62.6%) were discharged and 20 (10.5%) were still in hospital at the end of the study. Death was significantly associated with SBP (37.0% vs. 18.5%, p = 0.01), but no statistically significant association was found for the empirical antimicrobial treatment given (whether active or not against the isolated infectious agents). Other factors positively associated with death in univariate analysis (p < 0.10) were age (p = 0.05), ascitic neutrophil count (p = 0.04), involvement of E. coli (p = 0.007) and MDR bacteria (p = 0.07), whereas there was an inverse correlation between the involvement of coagulase-negative staphylococci and death (p = 0.01). No association was found between gender, the nosocomial or healthcare-related nature of the infection, the ascitic protein concentration, the use of fluoroquinolone prophylaxis, appropriate empirical therapy, or the total duration of antibiotic therapy and death.

In multivariate analysis, age (Odds Ratio (OR) 1.03 per year increment, Confidence Interval (95% CI): 1.00-1.06, p = 0.02), Child stage (OR 3.15 per stage increment, 95% CI: 1.57-6.32, p = 0.001) and MDR bacteria involvement (OR 2.30, 95% CI: 0.98-5.38, p = 0.055) were or tended to be significantly associated with death. These associations were similar when coagulase-negative staphylococci episodes were excluded.

## Discussion

Our study shows that: the spectrum of causative agents of SBP has changed since the EASL recommendations for the management of SBP [5], and thus that recommendations on antibiotics therapy should probably take into account these changes in bacterial epidemiology.

While Gram negative rods were the main infectious agents a few decades ago, and are still reported to be so in the most recent recommendations and reviews [6,12], our study confirmed that Gram positive cocci are now predominant, even when skin contaminants such as coagulase-negative staphylococci are excluded, or when the analysis is restricted to confirmed SBP or to non-nosocomial infections [10,13,14]. Moreover, even though the global implication of *E Coli* is declining slightly, the proportion of *E Coli* that produce betalactamase (including extended-spectrum betalactamase) is increasing, as is the proportion of *E Coli* with reduced susceptibility to fluoroquinolones, as already reported [9]. In addition, the proportion of enterococci infections is increasing, as already reported by us and others [10,13,14]. Enterococci were involved in nearly a quarter of the cases in our study, and these infections appear to be significantly associated with the use of quinolone prophylaxis, in particular in patients with confirmed SBP. Thus, quinolone prophylaxis, which is widely accepted and used by physicians in primary or secondary prevention of SBP [15], reduces the risk of infection, but favors the emergence of bacteria with natural or acquired resistance, such as enterococci, that are able to translocate [6,16]. This has to be kept in mind when considering fluoroquinolone prophylaxis, in particular for primary prophylaxis of SBP, which should be restricted to cirrhotic patients with low-protein ascites (<15 g/L), poor liver function and a fragile hemodynamic status, and to patients who are recovering from an episode of SBP who have a high risk of developing SBP [5,6].

Secondly, and in keeping with this evolving epidemiology, the currently recommended first-line antibiotic (third-generation cephalosporin alone) covered only one third of the infectious agents isolated from SBP in our study, and the two alternative choices (amoxicilline-clavulanate and fluoroquinolones) did not sufficiently increase the antimicrobial coverage. Even when only non-nosocomial, or non-health-care related, or non-coagulase-negative staphylococci infections were considered, no single therapy was able to offer antimicrobial coverage of more than 60%. This contrasts with the high clinical efficacy observed in previous reports, and particularly in clinical trials conducted between 1985 and 2000 [5,17,18]. The immediate use of antibiotic combinations for the empirical treatment of ascitic fluid infections should be considered, even though there is no strong clinical evidence to support this suggestion, nor any assessment of the impact on microbial resistance. The type of antibiotic combination may differ depending on the situation, as already suggested [19].

Amoxicillin clavulanate, cefotaxime, ceftriaxone, fluoroquinolones, teicoplanin and cotrimoxazole have satisfactory pharmacokinetics at significantly higher concentrations than the MICs of susceptible common pathogens in ascitic fluid [20-24]. Thus, as we observed in non-nosocomial episodes, combinations such as amoxicillin + third-generation cephalosporin, amoxicillin-clavulanate + cotrimoxazole or to a lesser extent amoxicillin-clavulanate + fluroquinolone (in patients without quinolone prophylaxis) may be of particular interest, considering their coverage and their limited risk of toxicity in cirrhotic patients. In nosocomial episodes (and particularly in SBP), teicoplanin could be an interesting association partner for betalactams (in particular piperacillin-tazobactam or penems). New broad-spectrum drugs such as ceftarolin should also be considered in specific studies.

Whether these suggested combinations are associated with a better clinical benefit than the currently recommended therapy has to be established. In the present study, although we observed a high in-hospital death rate (37% in SBP and 18.5% in bacterascites), we failed to demonstrate a survival benefit associated with the use of early appropriate antimicrobial therapy, even though we were unable to reliably adjust to the different associations used in current practice, the different timing and durations, and moreover the different management of comorbidities. The trend towards a higher risk of mortality associated with the involvement of MDR bacteria may not only be linked to greater therapeutic difficulties, but also highlight a surrogate marker of repeated exposure to antibiotics and of more frequent and longer hospitalizations. In addition, the fact that ascitic neutrophil counts were not systematically assessed and that antimicrobial therapy for confirmed SBP was not used systematically may have affected the results of the study, even though there was no clear difference between these unclassified episodes and the others. In addition, bacterascites must be considered a serious condition given the mortality rate we observed in patients with confirmed bacterascites (close to 20%), even though it is probably in part also a surrogate marker of advanced liver disease. Another potential limitation is that the statistical power may be insufficient, since the incidence of positive ascitic fluid culture (and of SBP) was lower than that observed a few years ago in the same area. Last, and more generally, microbial epidemiology may also vary from one region to another or from one ward to another [6].

## Conclusion

Physicians have to be aware of the limits of antimicrobial therapy. Recommended first-line treatments are at risk of becoming ineffective on the infectious agents involved, at least in culture-positive SBPs. However, they may still be effective in patients with negative ascitic fluid cultures. Quinolone prophylaxis, even though associated with a clinical benefit by lowering the frequency of infectious episodes, is also likely to increase the involvement of bacteria with acquired or natural resistance to quinolones, such as enterococci. Combinations such as amoxicillin + third-generation cephalosporin, or amoxicillin-clavulanate + cotrimoxazole, deserve to be considered in non-nosocomial episodes in patients with a severe clinical presentation, and in those with no biological improvement in the ascitic fluid after 48 hours of first-line therapy. Last, since the benefits and the risks of such proposals have to be established, there is a need not only for surveys but also for new clinical trials that include the newest broad-spectrum antibiotics conducted in the current epidemiological context.

## Competing interests

The authors declare that they have no competing interests.

## Authors’ contributions

LP, AnP, VdM and AM participated in the design of the study; YH, AP, IP, TH, BJ, CC, CR, Al, CN participated in the collection of the data and helped in their interpretation; LP, AnP, VdM and AM analyzed the data; LP, VdM, PC and AM drafted the manuscript. All authors read and approved the final manuscript.

## Pre-publication history

The pre-publication history for this paper can be accessed here:

http://www.biomedcentral.com/1471-2334/14/287/prepub
